# Development and validation of a flow cytometry antibody test for *Lawsonia intracellularis*

**DOI:** 10.3389/fimmu.2023.1145072

**Published:** 2023-03-21

**Authors:** Débora Zini Baldasso, João Antônio Guizzo, Cláudia Cerutti Dazzi, Gabriela Carolina Paraboni Frandoloso, César Feronato, Stephan von Berg, Roberto Maurício Carvalho Guedes, Heather Lynne Wilson, Luiz Carlos Kreutz, Rafael Frandoloso

**Affiliations:** ^1^ Laboratory of Microbiology and Advanced Immunology, Faculty of Veterinary Medicine, University of Passo Fundo, Passo Fundo, Brazil; ^2^ Section of Immunology, AFK Imunotech, Passo Fundo, Brazil; ^3^ Swine Technical Department, Merck Sharp & Dohme (MSD), São Paulo, Brazil; ^4^ Global Marketing Swine, Merck Sharp & Dohme (MSD), Animal Health, Munich, Germany; ^5^ Department of Veterinary Clinic and Surgery, Veterinary School, Universidade Federal de Minas Gerais, Belo Horizonte, Brazil; ^6^ Vaccine and Infectious Disease Organization (VIDO), University of Saskatchewan, Saskatoon, SK, Canada

**Keywords:** *Lawsonia intracellularis*, Ileitis, pig, diagnostic test, flow cytometry assay, serological antibody detection

## Abstract

*Lawsonia intracellularis* is the etiologic agent of porcine proliferative enteropathy (PPE), an inflammatory bowel disease with a major economic impact on the pig industry. The serological diagnosis of PPE can be performed using Blocking or Indirect ELISA, Immunoperoxidase Monolayer Assay (IPMA) and Indirect Fluorescence Antibody Test (IFAT). Here, we designed a most sophisticated immunological method for the detection of porcine anti-*L. intracellularis* IgGs, named Flow Cytometry Antibody Test - FCAT. This assay uses whole, live-attenuated *L. intracellularis* bacteria derived from a commercial vaccine. For the assay, we set up the optimal antigen concentration (10^6^ bacterium/assay), primary antibody dilution (1:100), time of incubation (20 min), antigen stability (15 days), precision (coefficient of variation - CV < 10%), reproducibility (CV ≤ 13%) and Receiver Operating Characteristic (ROC). When using a *cut-off* of >15.15% for FCAT, we determined that it showed a sensitivity of 98.8% and specificity of 100%. The rate of agreement with IPMA was 84.09% with a kappa index of 0.66. FCAT was used to screen 1,000 sera from non-vaccinated pigs housed in 22 different farms and we found that 730 pigs (73%) from 16 farms (72.7%) had *L. intracellularis* IgG. This high prevalence confirms that *L. intracellularis* is endemic on Brazilian pig farms. Finally, we determined that FCAT is an easy to perform diagnostic assay and we would highly recommend it for: i) seroepidemiological studies; ii) evaluation of infection dynamics; and iii) characterization of the humoral response profile induced by vaccines.

## 1 Introduction

*Lawsonia intracellularis* is an obligate intracellular Gram-negative bacterium that causes porcine proliferative enteropathy (PPE), an enteric disease with a major economic impact on the pig industry (1, 2). Pigs infected with *L. intracellularis* can develop two clinical forms of the disease depending on their previous immunological condition and the dose of bacteria ingested (3–5). The acute form, known as proliferative hemorrhagic enteropathy (6), affects mainly adult (> 4 months) animals (replacement gilts; growers) and, although clinically less frequent, it can cause high rates of mortality (up to 50%) (7, 8). The chronic form of PPE, in contrast, known as porcine proliferative enteropathy affects growing pigs (6 – 16 weeks of age) which has a low mortality rate, close to 1%, and might cause (but not always) a grey-green, semi-solid to liquid diarrhea (9).

Although the clinical presentations of the disease are very important, the silent (subclinical) course of the infection is most commonly observed on farms and, in this case, the presence of *L. intracellularis* can be demonstrated only by laboratory diagnosis assays as qPCR (if the bacteria are present) and serology (if the bacteria remain present for several months after infection/exposure) (10). The distribution of *L. intracellularis* in the global pig production system has been demonstrated and the prevalence of infected herds in Germany (91.7%), Denmark (95.8%), Spain (83.3%), France (79.2%), Netherlands (91.7%), United Kingdom (100%), Brazil (37%), China (77%), Canada (>50%) and Australia (100%) (10–14) illustrates the epidemiological importance of this microorganism. Now that pig farming is progressively reducing the use of antimicrobials as preventive measure, more prevalence of pathogenic bacteria in herds will be present.

The diagnosis of *L. intracellularis* can be confirmed by different assays, which can be used according to the type of biological sample collected. During post-mortem investigations, histological analysis complemented with Warthin-Starry silver staining (15), immunohistochemistry (16), and *in situ* hybridization (17) can be used to directly detect the bacteria in tissues. During *in vivo* monitoring, qPCR is the best technique to detect and determine the load of *L. intracellularis* in feces (4). ELISA (18), immunofluorescence antibody test (IFAT) (19), and immunoperoxidase monolayer assay (IPMA) (20) can be used to assess antibodies against the microorganism to establish whether the animals were exposed naturally (infection) or artificially [experimental challenges (controlled infection) and vaccination (Porcilis^®^ Ileitis and Enterisol^®^ Ileitis vaccines)].

Serological diagnosis is an excellent tool for assessing the kinetics of infection, the profile and duration of passive immunity, and the potency and duration of the vaccine-induced antibody response. Although these data can be easily obtained for other pathogens such as porcine circovirus type 2 and Influenza A virus, for which several commercial diagnostic kits with high sensitivity and specificity are available, this is not the case for *L. intracellularis*. For this microorganism, only two commercial ELISA kits (Blocking ELISA - Svanovir^®^ *L. intracellularis*/Ileitis-Ab, Sweden, and Indirect ELISA for research use only - Biostone™ Animal Health, USA) are available and whose sensitivity (Blocking ELISA) close to 72% is considered low. Furthermore, although it is possible to use IFAT and IPMA techniques for detecting anti-*L. intracellularis* IgG, in practice, few laboratories around the world offer these assays as a service, mainly due to the difficulty of cultivating *L. intracellularis*. Therefore, the use of serology to understand the infection dynamic or general sanitary condition of the farm for *L. intracellularis* is limited.

In order to overcome the limitations described above, in this study we used an innovative strategy to develop a new serological diagnostic test to detect antibodies against *L. intracellularis*. This test, named Flow Cytometry Antibody Test (FCAT), uses a live-attenuated strain of *L. intracellularis* as detecting antigen readily available from a commercial vaccine. FCAT has high sensitivity and specificity, is easy to perform, and can be promptly incorporated into the diagnostic routine of any specialized laboratory around the world.

## 2 Materials and methods

### 2.1 Statement of institutional animal care

All sera used in this study came from: i) other studies previously approved by the Ethics Committee for the Use of Animals in Research at the Universidade de Passo Fundo (CEUA no. 10/2020, 19/2020 & 20/2020); ii) routine of diagnosis of the AFK Imunotech Laboratory; iii) other studies previously approved by the Ethics Committee for the Use of Animals in Research at the Universidade Federal de Minas Gerais (CEUA n° 133/2018, 36/2016).

### 2.2 Antigen

The *in vitro* cultivation of *L. intracellularis* is extremely difficult, which limits the production of this bacterium to few laboratories in the world. Since our objective was to develop a serological assay that could be easily performed in any laboratory, we used the vaccine strain of *L. intracellularis* from Enterisol^®^ Ileitis (Boehringer Ingelheim), a live attenuated vaccine, as a source of antigen. This licensed vaccine is distributed worldwide, and all new batches of live antigen are qualified by the manufacturer, an ideal situation to guarantee antigen quality and ensure repeatability between antigens batches and laboratories that will perform this diagnosis.

### 2.3 Sera samples

A total of 246 serum samples obtained from pigs with different serological condition were used to determine the preliminary *cut-off* values of this flow cytometry antibody test (**Table 1** **, Phase I**). The second set of specimens included 1,200 serum samples collected between 2018 and 2021 from 32 farms located in Southern region of Brazil (Paraná, Santa Catarina and Rio Grande do Sul) (**Table 1** **, Phase II**); this panel of sera was used to analyze the ability of the established *cut-off* to identify conventional pigs with or without anti-*L. intracellularis* IgGs.

**Table 1:** Description of the sera used in this study.

Sera from	N° of Samples	Serological and molecular status of pigs at the time of blood collection	
		IgG anti-*L. intracellularis*	*L. intracellularis* excretion (feces)
Phase I – Preliminary *cut-off*			
Conventional pigs	38^A^*	Negative (IPMA)	Negative (qPCR)
Conventional pigs	40^B^**	NPE	Negative (qPCR)
Specific pathogen free (SPF) pigs	40^B^	Negative (ELISA)	Negative (qPCR)
SPF pigs vaccinated with Porcilis^®^ Ileitis - MSD	40^B^	Positive (ELISA)	Negative (qPCR)
SPF pigs vaccinated with Enterisol^®^ Ileitis - BI	20^B^	Positive (ELISA)	Negative (qPCR)
Conventional pigs experimentally infected pigs with *L. intracellularis*	20^A^*	Positive (IPMA)	Positive (qPCR)
Specificity			
SPF pigs vaccinated with *Glaesserella parasuis* (21)	8^C^	NPE	Negative (qPCR)
SPF pigs vaccinated with *Mycoplasma hyopneumoniae* (Safesui Mycoplasma - Ourofino)	8^B^	NPE	Negative (qPCR)
SPF pigs vaccinated with *Pasteurella multocida* A (Govaxx^®^ - Vaxxinova)	8^B^	NPE	Negative (qPCR)
SPF pigs vaccinated with *Salmonella* choleraesuis (Enterisol^®^ SC-54 - BI)	8^B^	NPE	Negative (qPCR)
SPF pigs vaccinated with *Escherichia coli* and *Clostridium perfringes* (Porcilis^®^ Coliclos - MSD)	8^B^	NPE	Negative (qPCR)
SPF pigs vaccinated with PCV2 (Porcilis^®^ PCV ID - MSD)	8^B^	NPE	Negative (qPCR)
Phase II – *Cut-off* evaluation			
Non-vaccinated conventional pigs	258^B^	NPE	Negative (qPCR)
Non-vaccinated conventional pigs	742^B^	NPE	Positive (qPCR)
Vaccinated (Porcilis^®^ Ileitis, MSD) conventional pigs	200^B^	NPE	NPE

Origin of the serum samples: A) Universidade Federal de Minas Gerais, prof. Roberto Guedes. Sera from pigs with 100 – 160 days old; B) AFK Imunotech (clinical trials). Sera from pigs with 63 – 120 days old; C) Study conducted by Ramos et al. (21). *) Sera previously titrated by IPMA. **) Sera from 150-day-old gilts from a closed genetic herd, with no clinical history of Ileitis. NPE) Samples not previously evaluated. SPF pigs were negative for the presence of *Glaesserella parasuis*, *Actinobacillus pleuropneumoniae*, *Pasteurella multocida* A & D, *Bordetella bronchispetica*, *Streptococcus suis*, *Mycoplasma hyopneumoniae*, *Lawsonia intracellularis*, Swine Influenza Virus A and PCV2.

### 2.4 Antigen stability

The stability of the reconstituted antigen was evaluated daily for a period of 30 days. Three parameters were analyzed by flow cytometry: i) absolute count of *L. intracellularis*; ii) bacterial morphology; and iii) antigenicity. For the first two analyses, a daily aliquot of the antigen was diluted 1:1,000 in filtered PBS pH 7.4 and acquired in the cytometer. The number of bacteria per microliter of acquired samples was calculated automatically by the cytometer which was equipped with flow sensor used for volumetric measurement. Therefore, to calculate the total number of bacteria per mL of the reconstituted vaccine, we used the following mathematical formula: number of events per μL × inverse of the dilution factor (1,000) × per 1,000 (to convert μL to mL). The morphometric analysis was conducted analyzing the parameters of Forward Scatter (FSC) vs Side Scatter (SSC) of the daily aliquot of the antigen. For the antigenicity analysis, 3 sera from pigs immunized with the Porcilis^®^ Ileitis vaccine were used; briefly, 10^6^ bacteria were incubated into the wells of a 96 well conical-bottom polystyrene plates (cat. # K30-6096V, Olen, China) together with 100 µL of porcine serum diluted 1:100 in filtered (0,22 µm, cat. # GPWP04700, Millipore, Ireland) PBS pH 7.4 containing 1% bovine serum albumin (cat. # A2153, Sigma-Aldrich, USA) (FACS buffer) for 1 hour at 37°C. After three washing steps with 200 μL of FACS buffer (plate centrifuged at 1,200 × g for 5 min; centrifuge 5810R Eppendorf, Germany), 100 μL of FACS buffer containing 1 μg of Goat anti-Porcine IgG(H+L)-PE (cat. # 6050-09, SouthernBiotech, USA) was added and incubated for 1 hour at 37°C. Then, the washing steps were repeated, and the bacterial pellet resuspended in 350 µL of PBS for analysis. All parameters were analyzed by Flow Cytometry using a FACSVerse Cytometer (Becton Dickinson, USA) equipped with a 405 nm violet laser, 488 nm blue laser, 640 nm red laser and flow sensor. A total of 100,000 events were acquired in P1 region and analyzed. Each sample was analyzed in triplicate.

### 2.5 Determination of the ideal concentration of *L. intracellularis* for the assay

To determine the optimal amount of antigen that would be used in the assay, three different concentrations (10^5^, 10^6^ and 10^7^) of *L. intracellularis* were evaluated. Each concentration was incubated with a panel of sera (n=5) with different titers of anti-*L. intracellularis* IgG as defined previously by the IPMA (**Table 1**): i) highly positive sera (titer between 1:960 and 1:1,920); ii) moderately positive sera (titer between 1:120 and 1:480); and iii) weakly positive sera (titer of 1:30). The immunostaining was performed as described above (subsection 2.3) and the ideal bacteria concentration was defined as the smallest number of bacteria that: i) allowed the statistical differentiation of three sera categories and ii) ensured enough bacteria to be analyzed after the washing steps.

### 2.6 Primary antibody working dilution

To define the optimal dilution of the primary antibody (porcine sera) different dilutions of a panel of highly positive (n=5, titer > 960), moderately positive (n=5, titer between 1:120 and 1:480, weakly positive (n=5, titer of 1:30) and negative (n=5) sera were analyzed. The dilutions evaluated were 1:50, 1:100 and 1:200 in a final volume of 100 µL. The immunostaining was performed as described above (subsection 2.3).

### 2.7 Evaluation of the incubation period of primary and secondary antibodies

To investigate the shortest incubation period necessary to guarantee the optimal interaction between the molecules involved in the assay, three incubation periods were evaluated: 60 min, 30 min and 20 min. These periods were evaluated for primary (10 different positive samples) and secondary (Goat Anti-Porcine IgG (H+L)-PE) antibodies. All incubations were performed at 37°C. The immunostaining was performed as described above (subsection 2.3).

### 2.8 Enzyme-linked immunosorbent assay - ELISA

A total of 100 samples (**Table 1**) were analyzed for the presence of anti-*L. intracellularis* antibodies using a commercial blocking ELISA Kit (SVANOVIR^®^, Boehringer Ingelheim Svanova, Sweden). The protocol was performed following the manufacturer’s recommendations.

### 2.9 Immunoperoxidase monolayer assay

The immunoperoxidase monolayer assay (IPMA) was used to detect porcine anti-*L. intracellularis* IgG. This assay was carried out by the laboratory of Prof. Roberto Guedes (Universidade Federal de Minas Gerais), according to the methodology previously described (20).

### 2.10 Flow cytometry antibody test

**Figure 1:**
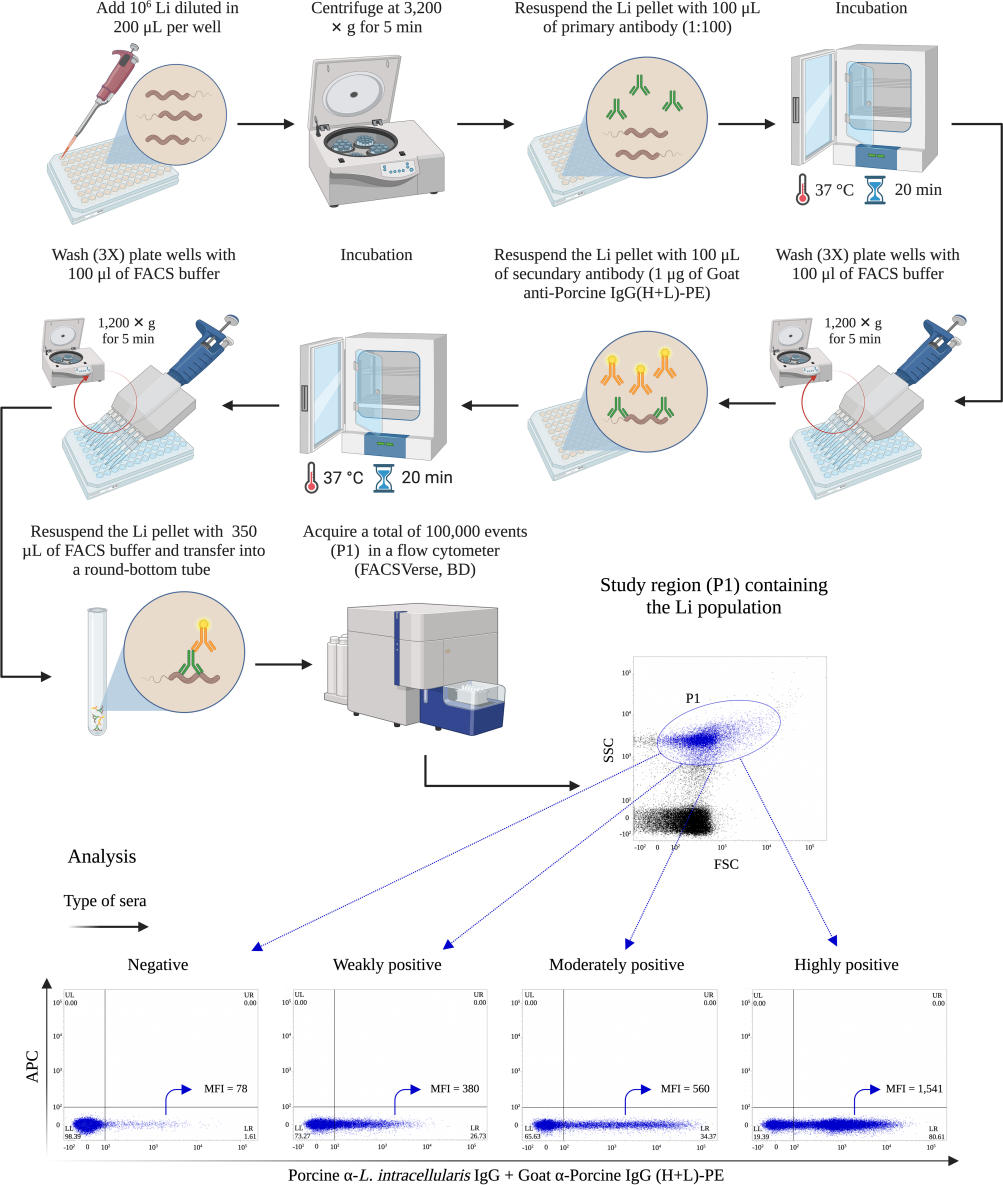
Quick protocol of flow cytometry antibody test for *Lawsonia intracellularis*. The brief protocol of immunostaining and cytometric analysis for the detection of porcine anti-*L. intracellularis* (Li) IgG. MFI, Mean Fluorescence Intensity. This figure was created with BioRender.com.

A total of 10^6^ *L. intracellularis* suspended in 200 µL was added per well of a 96 well conical bottom polystyrene plates. The plate was centrifuged (3,200 × g, 5 min) and the bacterial pellet was resuspended in 100 µL of porcine serum diluted 1:100 in FACS buffer and incubated at 37°C during 20 min. After three washing steps with 200 μL of FACS buffer (step of centrifugation at 1,200 × g for 5 min between washes), 100 μL of FACS buffer containing 1 μg of Goat Anti-Porcine IgG(H+L)-PE was added and incubated for 20 min at 37°C. Then, the bacteria were washed 3 times, resuspended in 350 µL of FACS buffer and transferred into a round-bottom tube (Falcon^®^, cat. # 352052). A total of 100,000 events were acquired in a FACSVerse cytometer. The bacterial population was initially identified through a dot plot crossing the FSC and SSC parameters; where the study region, designated P1, was established. Subsequently, a second dot plot was created, in this case, crossing the FL-2 (Phycoerythrin, PE) and FL-4 (Allophycocyanin, APC) channels and having P1 as the study region. The events considered positive were those enclosed within the quadrant representative of PE (low right). The results are expressed as the percentage of positive bacteria in relation to the total bacteria contained in the P1 region. The step-by-step execution of this diagnosis as well as the analysis strategy are illustrated in **Figure 1**.

### 2.11 Receiver operating characteristic

The determination of the *cut-off* was performed using a panel of 198 porcine sera (**Table 1** **)**. Briefly, sera from 78 animals known to be negative for anti-*Lawsonia intracellularis* IgG and molecularly (qPCR) negative for *L. intracellularis* were obtained. We also obtained sera from 40 animals from a genetic nucleus herd with no history of Ileitis; these animals were molecularly negative for *L. intracellularis*; collectively sera from a total of 118 negative animals. We used sera from 80 animals serologically positive for *L. intracellularis*; of these total, 60 SPF pigs were vaccinated (qPCR negative), and 20 were experimentally infected (and consequently qPCR-positive). Therefore, the *cut-off* calculation, performed using the Receiver Operating Characteristic (ROC) Curve, included 118 negative and 80 positive animals. All sera were tested by this new assay and the results were used to generate a Receiver Operating Characteristic (ROC) Curve. The selected *cut-off* was based on the sensitivity and specificity values. The ROC Curve analysis was performed using Graph Pad Prism Software Version 9.2.0.

### 2.12 Specificity

The specificity analysis was conducted to evaluate the ability of this assay to identify IgG specifically to *L. intracellularis*, which is the analyte of interest. The analysis was performed as recommended by Selliah, Eck (22). Briefly, a panel of 48 sera (**Table 1** **, Specificity)** from SPF pigs immunized with *Glaesserella parasuis* (bacterin prepared with SV7 (21), n=8), *Mycoplasma hyopneumoniae* (Safesui Mycoplasma, Ourofino, n=8), *Pasteurella multocida* A (Bacterin formulated with a Brazilian clinical strain of *P. multocida* A, *pfhA* ^+^, Vaxxinova, n=8), *Salmonella enterica*, serovar Choleraesuis (Enterisol^®^ SC-54 vaccine, Boehringer Ingelheim, n=8), *Clostridium perfringes* type C and *Escherichia coli* (Porcilis^®^ Coliclos, MSD, n=8) and Porcine circovirus type 2 (Porcilis^®^ PCV ID, MSD, n=8) were assessed using the protocol described in subsection 2.8.

### 2.13 Precision and reproducibility

Precision is one of the most critical parameters in flow cytometry assay (22). In order to know the intra-assay precision (precision), samples from 3 positive pigs (vaccinated with Porcilis^®^ Ileitis) and negative SPF pigs were tested twice under the same conditions by a single analyst. With the values obtained from each sample, the mean and coefficient of variation were calculated. For reproducibility analysis (inter-assay precision) the same samples were analyzed by 2 different analysts on 2 different days. Again, with the values obtained from each sample, the mean and the coefficient of variation were calculated. The accuracy acceptance criterion was established by accepting a coefficient of variation between the analyses of the same analyst or between the analysts of a maximum of 25%.

### 2.14 Agreement analysis between FCAT and IPMA techniques

To analyze the level of agreement between the FCAT and IPMA, we used a panel of sera from two experimental groups: G1) pigs (n = 8) immunized with the Porcilis^®^ Ileitis vaccine at 21 days of life and G2) pigs (n = 8) inoculated with PBS. Serum samples were collected prior to vaccination (D0) and at the following post-vaccination times: D7, D14, D21, D28 and D55 contributing to 88 sera for comparative purposes. Agreement analysis (Kappa) was performed as described by Landis and Koch (23).

### 2.15 Statistical analysis

All data were analyzed using GraphPad Prism™ (GraphPad Software, San Diego, California, USA). One or two-way ANOVA with Tukey’s multiple comparisons test were used to assess significance between the different variables analyzed in this study. The specific test used in each analysis, as well as the significance, is indicated in the figure legends.

## 3 Results

### 3.1 Antigen stability

**Figure 2:**
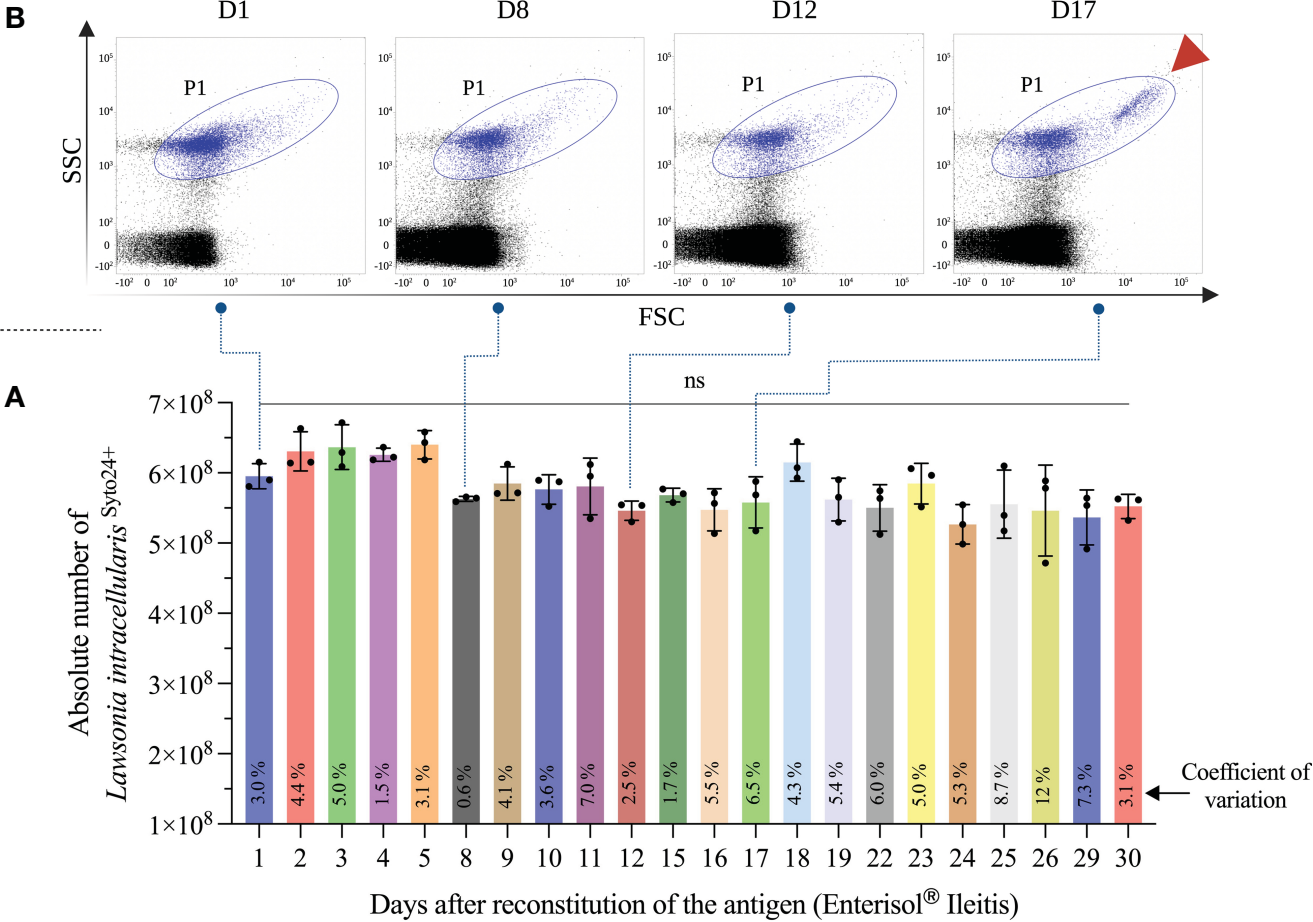
Antigen stability analysis. An aliquot of Enterisol^®^ Ileitis vaccine reconstituted and stored at 4-8°C was analyzed daily by flow cytometry. The aliquot diluted 1:1,000 was analyzed in triplicate and the mean of *L. intracellularis* absolute count plus standard deviation is shown in “**(A)**”. The coefficient of variation of each quantification is represented at the foot of the bars. The morphological characteristic of the bacterial population at 4 different times is illustrated in “**(B)**”. The subpopulation of *L. intracellularis* with greatest size and complexity is indicated with the arrowhead highlighted in red. Statistical comparison was performed using one-way ANOVA with Tukey’s multiple comparisons test and no significant (ns) differences were found between the moments compared.

**Figure 3:**
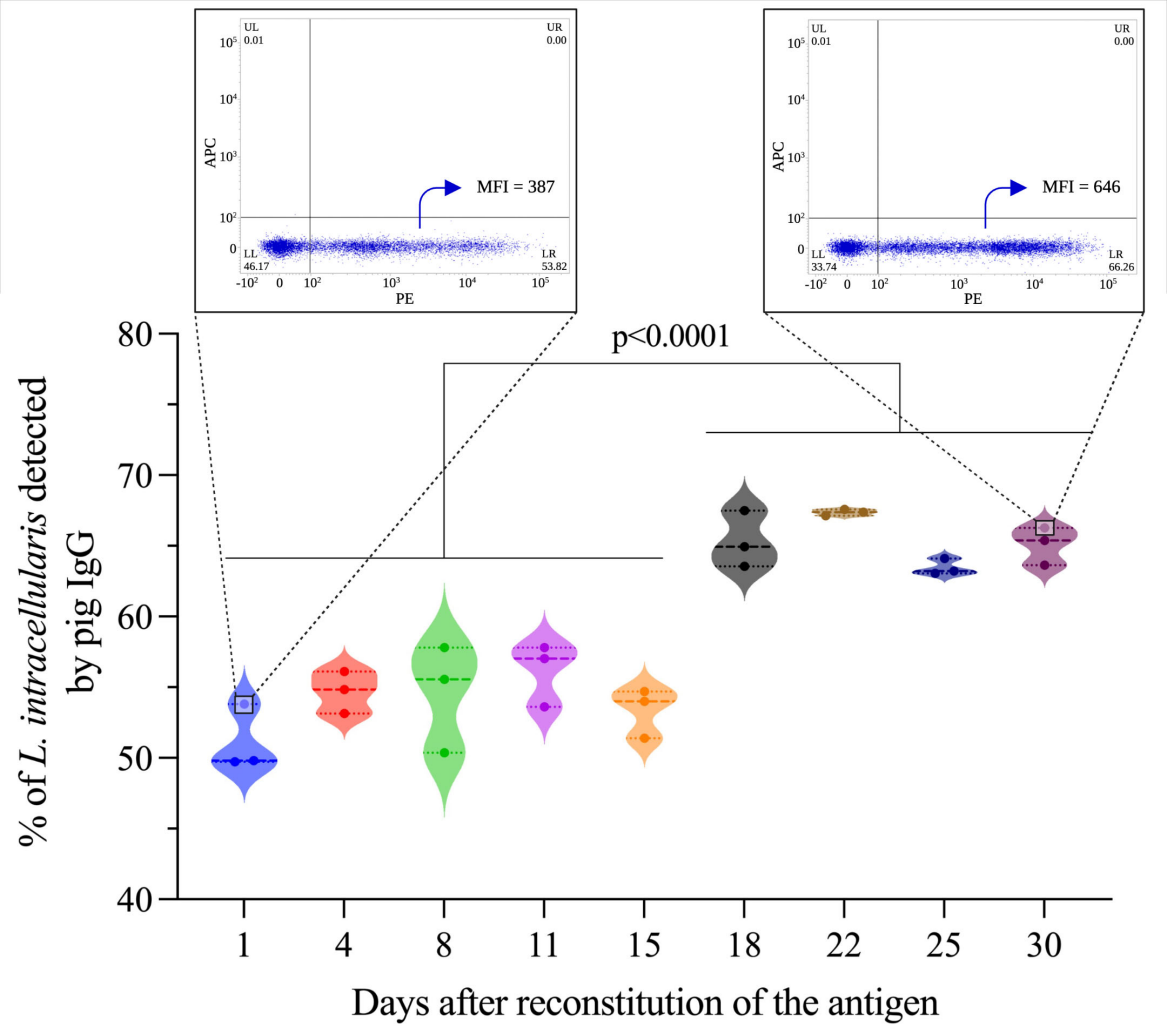
Antigenicity analysis of the reconstituted antigen. This analysis was performed by flow cytometry using sera (n=3) from pigs immunized with the Porcilis^®^ Ileitis vaccine. The results express the percentage of *L. intracellularis* bacteria (total population containing 10^6^ bacteria) containing antibodies (IgGs) surface. Statistical comparison was performed using one-way ANOVA with Tukey’s multiple comparisons, and statistical differences are represented in the figure. Dot plots showing antigenic differences between time D1 and D30 are highlighted in zoom format within the figure.

*L. intracellularis* obtained from the attenuated vaccine Enterisol^®^ Ileitis was used as a diagnostic antigen. The antigen used in our study is destined to immunizing pigs and should be immediately used upon reconstitution. In the test designed here, the amount of antigen used in each assay is low and a single reconstituted bottle contains enough antigen to be used along a few weeks. Thus, the stability and quality of the reconstituted and refrigerated (4 – 8°C) antigen was analyzed daily along a 30 days period. During this time, changes in the morphology of the bacterial population (analyzed in the P1 region) were observed after 15 days of antigen storage (**Figure 2B**); a subpopulation of larger and more complex *L. intracellularis* was clearly detected, which may represent loss of bacteria cell wall integrity. Although we observed morphological changes in the bacterial population, the number of bacteria detected at each time point was similar with no significant reduction at any time (**Figure 2A**). The antigenicity analysis revealed that antigen stability follows the same trend as bacteria reconstituted for more than two weeks were significantly more antigenic compared to previous time points (**Figure 3**), which indicated that storage time alters the antigenic characteristics of the reconstituted antigen; thus, for all assays the reconstituted antigen was used for 15 days only.

### 3.2 Determination of the ideal concentration of *L. intracellularis* for the assay

**Figure 4:**
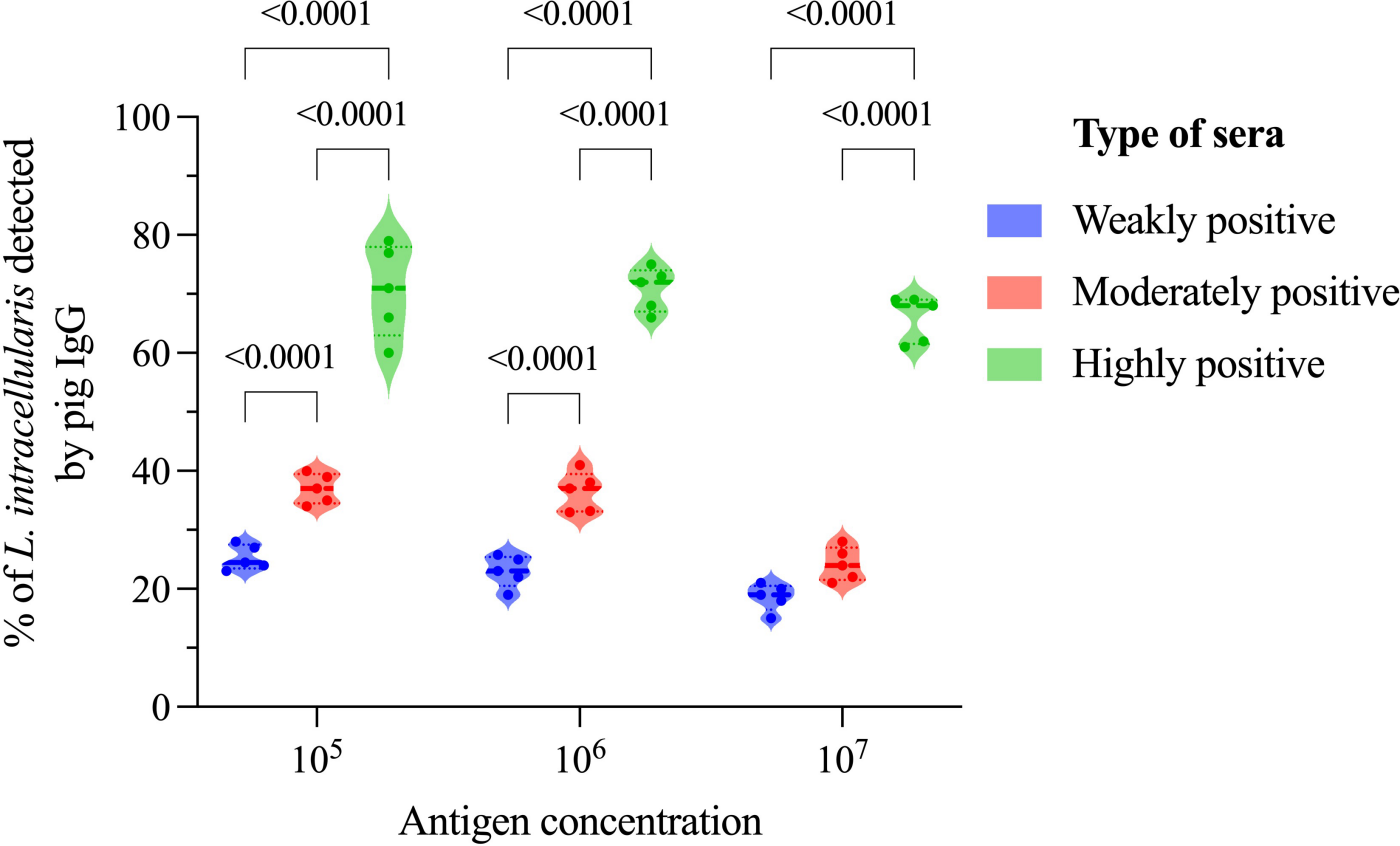
Impact of antigen concentration on anti-*L. intracellularis* IgG detection. In this analysis 3 different concentrations of antigens (10^5^, 10^6^ and 10^7^) and 3 different categories of sera (weakly, moderately, and highly positive for *L. intracellularis*) were analyzed by flow cytometry after immunostaining. Statistical comparison was performed using two-way ANOVA with Tukey’s multiple comparisons. Statistical differences are indicated in the figure.

The amount of antigen used in a diagnostic test must be evaluated with two objectives: i) functionally (to ensure maximum resolution of the test) and ii) economically (antigen saving). Taking these premises into account, we analyzed 3 concentrations of antigens (10^5^, 10^6^, and 10^7^) immunostained with 3 categories of sera, weakly, moderately, and highly positive as determined by IPMA. As illustrated in **Figure 4**, regardless of the bacterial concentration it is possible to differentiate the 3 serum categories, except for the concentration of 10^7^, in which weakly and moderately positive sera could not be statistically differentiated. There is a reduction in the percentage of detection of *L. intracellularis* between concentrations of 10^6^ and 10^7^, when using weakly and moderately positive sera (**Figure 4**). Additionally, we noticed that the number of bacteria acquired from immunostaining using 10^5^ bacteria was lower (long acquisition period) compared to 10^6^ and 10^7^, indicating that part of the bacteria was lost during the washing steps; therefore, the concentration of 10^6^ was defined as the ideal concentration of antigen.

### 3.3 Assessment of primary antibody dilution

**Figure 5:**
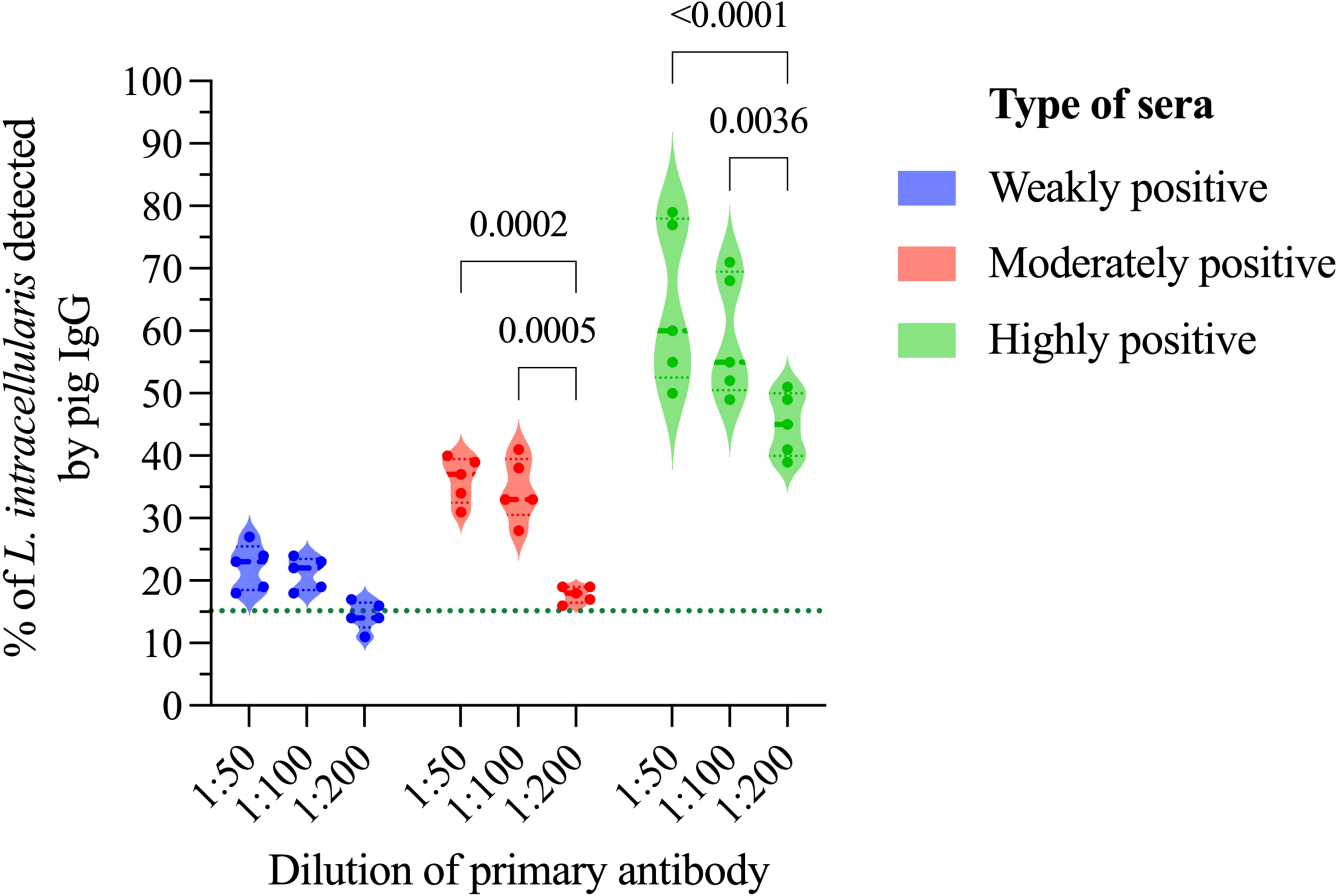
Primary antibody dilution and its impact on serology interpretation. In this analysis 3 different dilution (1:50, 1:100 and 1:200) of primary antibody (pig sera) classified in 3 different categories (weakly, moderately, and highly positive for *L. intracellularis*) were analyzed by flow cytometry after immunostaining. Statistical comparison was performed using two-way ANOVA with Tukey’s multiple comparisons. Statistical differences are indicated in the figure.

The dilution rate of the serum (primary antibody) is essential in any diagnostic test and should be set to mitigate false positive results, usually associated with polyreactive IgGs. Three serum dilutions were analyzed; 1:50, 1:100 and 1:200. We observed that there was no reduction in the percentage of *L. intracellularis* associated to porcine IgGs between the 1:50 and 1:100 dilutions, regardless of the serum category. On the other hand, the percentage of *L. intracellularis* with associated IgGs was significantly lower (p < 0.0001) when sera diluted 1:200 were used, and the number of sera considered positive (*cut-off* set at 15.15% as described in the section 3.5) at lower dilutions (5/5) decreased when the weakly positive sera category was tested (2/5) (**Figure 5**). Therefore, the dilution of the primary serum was set at 1:100.

### 3.4 Selection of the best antibody incubation time

**Figure 6:**
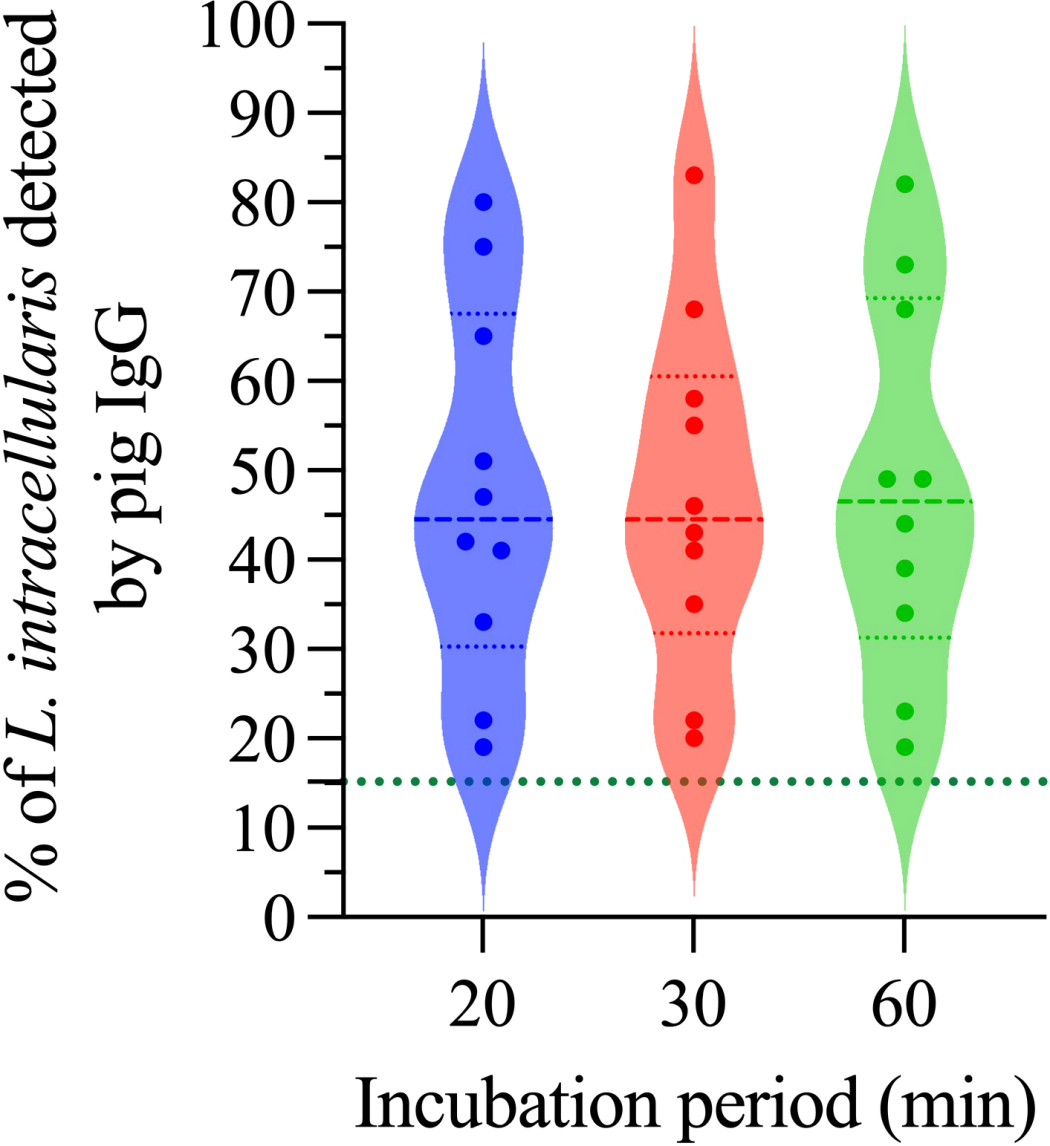
Outcome of incubation time on the detection of anti-*L. intracellularis* IgG. A total of 10 pig sera positive for IgG anti-*L. intracellularis* were included in this analysis. Pig sera were incubated for 20, 30 and 60 minutes with 10^6^ *L. intracellularis* at 37°C. Subsequently, the samples were incubated with the secondary antibody for the same times. Statistical comparison was performed using one-way ANOVA with Tukey’s multiple comparisons. No statistical differences (ns) were observed between the different times analyzed.

Evaluating antibody incubation time (primary and secondary) is essential in any diagnostic test under development; in general, it is always recommended to select the shortest time, as long as there are no changes in the final result of the diagnosis. Three incubation times (20, 30 and 60 min) for both the primary (pig IgG anti-*L. intracellularis*) and secondary (Goat anti-Porcine IgG-PE) antibody were tested. As illustrated in **Figure 6**, the mean percentage of *L. intracellularis* with associated IgGs at the 3 different incubation times was 47.5%, 47.1% and 48.0%; additionally, the same dispersion profile of the results was observed, and no significant differences were found between the analyzed times. In view of these results, an incubation time of 20 min was selected for the primary and secondary antibodies.

### 3.5 Specificity and sensitivity of the flow cytometry antibody test

**Figure 7:**
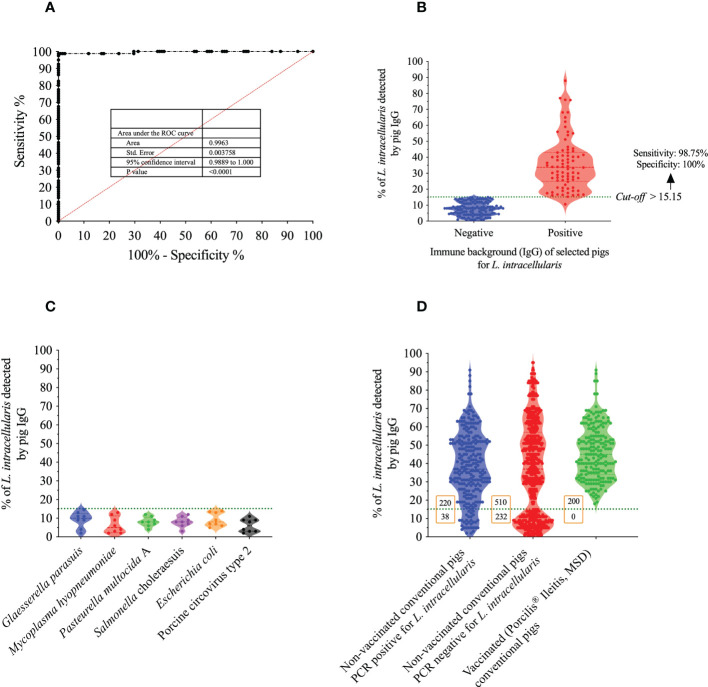
Basic immunological characteristics of the FCAT method. **(A)** Receiver Operating Characteristic (ROC) analysis. **(B)** Representation of the percentage values (dispersion) of *L. intracellularis* recognition of the samples used to define the *cut-off* of the FCAT test. The sensitivity and specificity of the test using the *cut-off* of > 15.15% are described in the figure. **(C)** FCAT specificity analysis using sera from pigs immunized with other pathogens. **(D)** Analysis of clinical samples collected from pigs with different immunological backgrounds against *L. intracellularis*.

Once the definition of all parameters and conditions of the Flow Cytometry Antibody Test (FCAT) was concluded, a wide panel of swine sera was used (**Table 1** **, Phase I**) aiming to find the *cut-off* value capable of truly differentiating a positive from a negative serum sample. For this purpose, a ROC curve analysis was conducted using 118 serum samples negative and 80 serum samples positive to *L. intracellularis* antibodies (**Figure 7A**). The ROC curve analysis of the FCAT data generated paired estimates of relative sensitivity and relative specificity at different *cut-off* values. A *cut-off* of 15.15% (percentage of *L. intracellularis* with associated IgG) was recommended; and at this *cut-off* value, the relative sensitivity and specificity estimates were 98.8% [95% confidence interval (CI) = 93.2% to 99.9%] and 100% (95% CI = 96.9% to 100%), respectively. The ROC curve had an Area Under the Curve (AUC) value of 0.9963 (95% CI = 0.99 to 1.0), which indicated a high level of accuracy for this FCAT (**Figure 7B**). Thereafter, using the established *cut-off* value, a second panel consisting of 48 sera (**Table 1** **, Specificity**) was tested on the FCAT. As illustrated in **Figure 7C**, none of the sera reached the *cut-off* of 15.15%; therefore, all sera were considered negative for *L. intracellullaris*. This result demonstrates that there is no cross-reactivity between *L. intracellularis* and *G. parasuis*, *M. hyopneumoniae*, *P. multocida* A, *Salmonella enterica*, serovar Choleraesuis, *E. coli*, *C. perfringes* type C and Porcine circovirus type 2. Finally, a third panel of sera (n=1,200, **Table 1**, **Phase II**) from: i) non-vaccinated conventional pigs [*L. intracellularis* (qPCR) negative feces]; ii) non-vaccinated conventional pigs [feces positive for *L. intracellularis* (qPCR)]; and iii) Vaccinated (Porcilis^®^ Ileitis, MSD) conventional pigs, was evaluated in the FCAT. As shown in **Figure 7D**, all animals vaccinated with the Porcilis^®^ Ileitis vaccine developed antibodies against *L. intracellularis* and were therefore considered positive. Similarly, 85.3% of the animals that were shedding *L. intracellularis* in the feces had anti-*L. intracellularis* IgG at a level higher than the FCAT *cut-off*; and 68.7% of the animals that were not shedding *L. intracellularis* at the time of blood collection had previous contact with *L. intracellularis*.

### 3.6 Precision and reproducibility of the FCAT

The precision (intra-assay) and reproducibility (inter-assay) analysis were performed by two different analysts (A and B). As described in the **Table 2**, the coefficient of variation (CV) of the samples analyzed in triplicate by each of the analysts was always less than 10%, indicating that analysts performed the immunostaining and acquisition of samples with precision. Furthermore, when the same samples were repeated by the same analyst on the following day, a CV of less than 10% was again observed. It is important to highlight that the results of immunostaining performed by the two analysts varied little, with the smallest variation being 0.9% and the maximum 13%; these data indicate that FCAT has high accuracy.

**Table 2:** Precision and reproducibility analysis of pig serum by different analyst at two different days.

Day	Serum	% of *L. intracellularis* detected by pig IgG			
		Analyst A		Analyst B	
		Mean	%CV	Mean	%CV
01	#01	10.3	6.2	11.1	6.0
	#02	12.2	1.2	12.7	3.8
	#03	14.7	2.3	12.8	2.5
	#04	41.4	7.2	39.1	7.1
	#05	36.7	7.2	37.2	4.5
	#06	52.3	3.4	57.5	2.8
02	#01	10.6	6.8	10.8	8.3
	#02	11.3	2.5	11.9	4.5
	#03	13.1	3.6	13.4	2.3
	#04	40.0	4.3	40.8	6.9
	#05	39.0	6.2	38.5	4.6
	#06	54.8	3.3	55.4	1.6

### 3.7 Comparison between FCAT and IPMA techniques

To analyze the level of agreement between FCAT and IPMA, sera collected from two groups of pigs (vaccinated and unvaccinated) at different post-vaccination periods were analyzed by these two methodologies in two different laboratories (FCTA conducted at the Frandoloso’s lab and IPMA conducted at Guedes’s lab). To avoid any bias, the analysis was blind, and the history of the sera was not revealed to the analysts. As described in **Table 3**, out of the total number of samples tested (n=88), 74 had the same result in both methodologies, which represents a direct agreement of 84.09%. Using all the data (samples with the same and different results according to the technique used) the Kappa index was generated resulting in a general agreement of 0.66 ± 0.08 (95% CI = 0.50 to 0.82) (**Table 4**), which represent a substantial agreement taken into consideration the Kappa scale (0 to 1).

**Table 3:** Serological evolution of pigs immunized or non-immunized pigs with *Lawsonia intracellularis*.

Groups	Assay	Number of positive animals/total number of animals evaluated					
		Experimental days					
		D0	D7	D14	D21	D35	D55*
G1 – Vaccinated	FCAT	0/8	4/8	6/8	8/8	8/8	4/4
	IPMA	1/8	4/8	4/8	8/8	8/8	4/4
G2 – Non-vaccinated	FCAT	0/8	0/8	0/8	0/8	0/8	0/4
	IPMA	0/8	2/8	4/8	1/8	0/8	0/4

*At D55, only 4 animals from each group were analyzed, since the others were sacrificed at previous moments for the analysis of other parameters (data not shown).

The presence of anti-*L. intracellularis* IgG in pig’s serum was evaluated by FCAT and IPMA.

**Table 4:** Matrix used to calculate the Kappa index.

	FCAT			
		Positive	Negative	Total
IPMA	Positive	26	10	36
	Negative	4	48	52
	Total	30	58	**88**

The calculation was performed online using the “Quantify Agreement with Kappa” tool from the GraphPad by Dotmatics website.

Considering that the pigs used in this experiment were Specific Pathogen Free and kept in a controlled facility (BSL-2) throughout the study, our expectation was that all animals, regardless of the diagnostic technique, would be negative for *L. intracellularis*. As illustrated in **Table 3**, we observed that a considerable number of pigs from group G2 (non-vaccinated) were positive for anti-*L. intracellularis* IgG as determined by the IPMA day (D) 7 (n=2, 25%), D14 (n=4, 50%) and D21 (n=1, 12.5%) post inoculation with PBS (**Table 3**). We understand that these IPMA results are false positives, and this interpretation is supported by two observations: i) samples collected from the same pigs at days D35 and D55 were negative by IPMA (**Table 3**); ii) all sera samples from this group were negative for anti-*L. intracellularis* IgG by FCAT.

The sensitivity to specifically detect the analytical, in our case the anti-*L. intracellularis* IgG during the genesis of humoral response induced by vaccination is one of the most important features. We noticed that both techniques are very similar in terms of sensitivity; however, FCAT standing out slightly over IPMA on D14, at that moment the FCAT classified more animals as positive than the IPMA.

## 4 Discussion

*Lawsonia intracellularis* is a complex intracellular bacterium ubiquitously found in the pig production system worldwide (11–13, 24). This microorganism is the causative agent of PPE which is considered one of most important enteric diseases of pigs in the growing and finishing phases. The presence of uncontrolled *L. intracellularis* infection in pigs has significant economic impacts due to its negative effect on daily gain, feed conversion rate and mortality (4, 25–27).

Currently, the control of PPE can be performed satisfactorily by using licensed vaccines, and in this line, three antigen delivery platforms are available to immunize pigs. Boehringer Ingelheim commercializes the Enterisol^®^ Ileitis vaccine, which is based on an attenuated strain of *L. intracellularis* and whose administration is carried out through the drinking water or by directly administration into the oral cavity of piglets. In parallel, MSD Animal Health has two inactivated vaccines, which can be administered intramuscularly (Porcilis^®^ Ileitis & Porcilis^®^ Lawsonia) and intradermally (Porcilis^®^ Lawsonia ID). The effectiveness of these vaccines has already been scientifically demonstrated (28–31) and their use in pig production is a valuable tool for the progressive reduction of antimicrobial usage as a measure to prevent this disease (32). Additionally, it is important to recognize that vaccines applied by the oral and intradermal routes are still scarce in pigs (and in mammals in general). Due to their demonstrated efficacy, they represent two platforms that should be priority for the veterinary pharmaceuticals industry. These vaccines are safe from the point of view of application (without needles, and therefore mitigate iatrogenic transmission of pathogens) and painless, which contemplates one of the premises of animal welfare.

During infection, *L. intracellularis* stimulates B lymphocytes to produce mucosal (IgA) (33) and systemic antibodies (IgG) (34). The immunization using inactivated-based vaccines consistently induces systemic IgG (28–30), a feature that is also observed in animals immunized with the attenuated vaccine (34). Therefore, antibodies (IgG) are a valuable marker to demonstrate the circulation of the agent in the farm, or even to monitor the antibody response after the application of vaccines.

Since the negative impact of *L. intracellularis* infection for pig production is unquestionable, in this study we present a new method of serological diagnosis for *L. intracellularis*, named Flow Cytometry Antibody Test. Flow cytometry has been used to quantify the percentage of eukaryotic cells which had taken up or were associated with fluorescent *L. intracellularis* (35). As described in **Table 5**, there are different diagnostic tests available on the market for the detection of anti-*L. intracellularis* IgG, which differ regarding sensitivity, specificity, execution time and complexity. Among these tests, the FCAT stands out in all parameters; the method is highly sensitive (98.8%), specific (100%) and takes approximately only 1:15 hours to run, which represents a time saving compared to Blocking ELISA, Indirect ELISA, IPMA and IFAT of 47%, 8%, 33% and 12%, respectively. Although the specificity of IPMA and FCAT are the same (100%), the sensitivity of the FCAT method (98.8%) is much higher than IPMA (89%), IFAT (91%) and Blocking ELISA (72%) (**Table 5**). In practical terms it represents a greater ability to detect truly positive samples even if they contain low levels of antibodies to the bacterium.

**Table 5:** Types and main characteristics of available serological assays for *L. intracellularis*.

Characteristics	Immunoassays for *Lawsonia intracellularis*				
	Blocking ELISA	Indirect ELISA	IPMA	IFAT	FCAT
Sensitivity	72%	NA*	89%	91%	98.8%
Specificity	93%	NA	100%	97%	100%
Time of execution	2:20 h	1:25 h	2:00 h	1:30 h	1:15 h
Reference	(18)	(36)	(20)	(37)	This study

*Not available.

**Figure 8:**
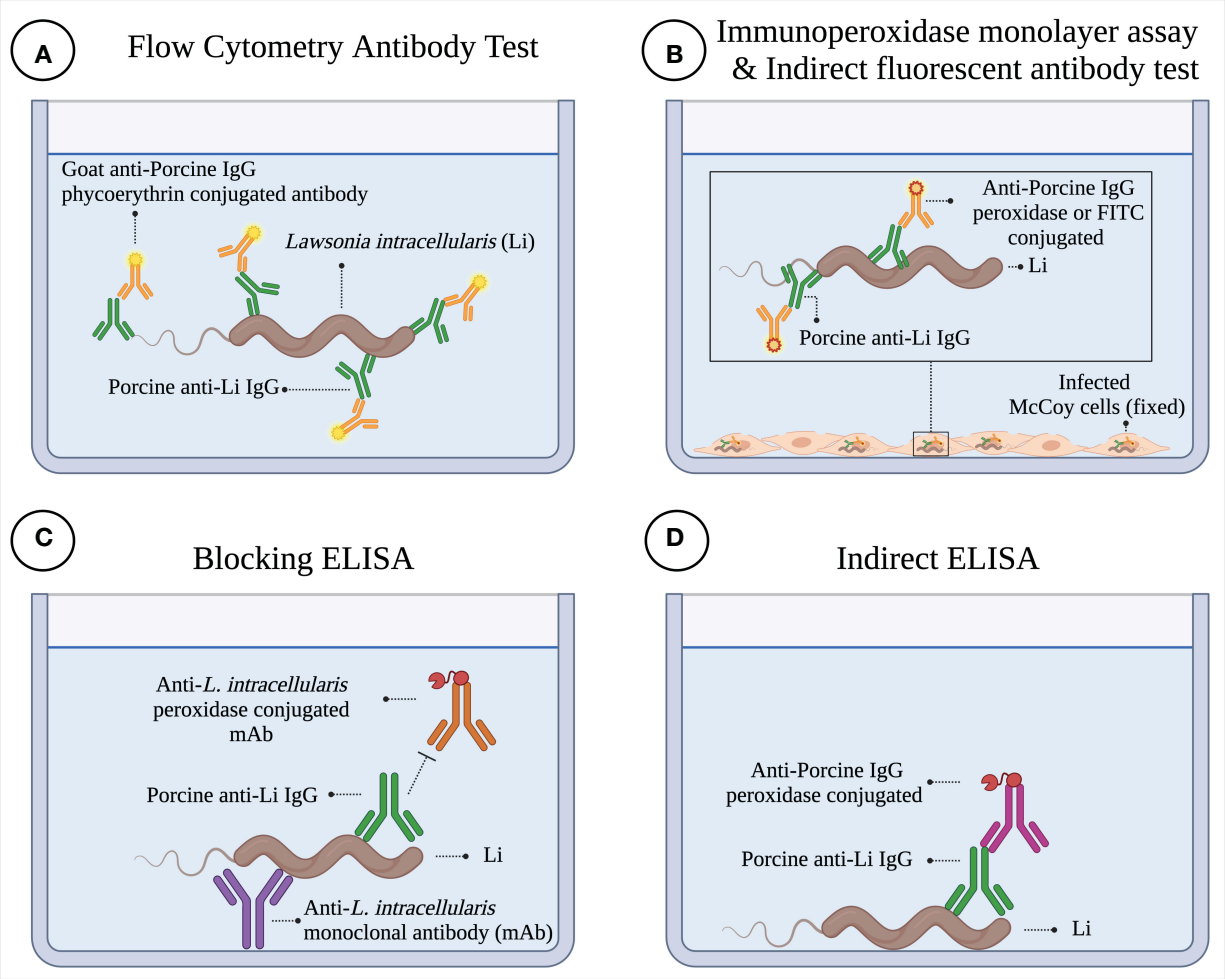
Illustration of different serological diagnostic tests for *Lawsonia intracellularis*. **(A)** Flow Cytometry Antibody Test. In this assay, porcine antibodies interact with *L. intracellularis* floating in the well of the plate. The binding of specific antibodies to *L. intracellularis* is demonstrated with a phycoerythrin-conjugated antibody specific for porcine IgG. The test is read on the flow cytometer. **(B)** Immunoperoxidase Monolayer Assay (IPMA) & Indirect Fluorescent Antibody Test (IFAT). In these assays, cells (i.e., McCoy cells) infected with *L. intracellularis* are chemically fixed with acetone-methanol, which promotes a structural change in the cell (cytoplasmic membrane damage, as indicated in figure). This damage is essential for porcine antibodies to interact with *L. intracellularis*. The presence of antibodies bound to *L. intracellularis* is demonstrated by a peroxidase conjugate antibody plus chromogen (3-amino-9-ethyl-carbazole) solution (IPMA). In the case of IFAT, a fluorescein isothiocyanate conjugated antibody is used to reveal the presence of porcine IgG bound to the pathogen. Plates are read under an inverted microscope. **(C)** Blocking ELISA. In this ELISA, a monoclonal antibody bound to the ELISA plate is used to capture *L. intracellularis*. Porcine antibodies compete with the peroxidase-conjugated monoclonal antibody for a specific epitope present on a surface antigen of *L. intracellularis*. The presence of porcine antibodies to *L. intracellularis* is determined by the potential to reduce or inhibit binding of the monoclonal antibody and, therefore, the enzymatic reaction associated with the competing conjugated antibody. **(D)** ELISA Indirect. In this ELISA, *L. intracellularis* is immobilized directly on the ELISA plate; and for this reason, a considerable surface area of the antigen is not accessible to antibodies (physical masking of antigens). Porcine antibodies that recognize *L. intracellularis* are detected with a peroxidase-conjugated anti-porcine IgG antibody. This figure was created with BioRender.com.

The reason why FCAT is superior to other tests is related to the basic characteristics of the assay, such as: i) live *L. intracellularis* is used in the test (the antigen source for this test is available globally – Enterisol^®^ Ileitis, BI); ii) the antigen is not chemically (acetone and methanol) treated during the test [all antigens (protein lipids and saccharides) remain native]; iii) the entire surface of the antigen is accessible to antibodies (increases the chance of antibodies specifically binding to the surface of *L. intracellularis*); and iv) the fluorescence reading is performed automatically by Flow Cytometry Equipment, and therefore, mitigates human errors (e.g. subjective counting) (**Figure 8A**). In contrast to FCAT, in the case of IPMA and IFAT, the cells are fixed with acetone and methanol, which promotes disruption of the cytoplasmic membrane of infected eukaryotic cells and might reduce the antigenic quality of the antigens (38) (**Figure 8B**). Additionally, in the case of IPMA, as the reading is performed manually with an inverted microscope, and as endogenous cell-derived peroxidases might be present on the assay, some false positive results can be expected (39). In this line, in our study, when analyzing sera from SPF piglets that were truly negative for *L. intracellularis*, we observed false positive results at four different times by the IPMA technique (**Table 3**); and, therefore, considering its biological limitation, the use of IPMA in the certification of negative farms for *L. intracellularis* should be carried out with caution.

In addition to the two mentioned techniques, the detection of porcine anti-*L. intracellularis* antibodies can be performed by ELISA. As illustrated in **Figure 8C**, the blocking ELISA is based on i) monoclonal antibody-coated wells for capturing of cell-cultured antigen and ii) utilizes peroxidase-conjugated monoclonal antibodies as competitive antibodies. In this case, one of the disadvantages of this method in relation to FCAT is that the blocking of the conjugated monoclonal antibody is restricted to a single epitope, and therefore, for an animal to be diagnosed as positive, it must produce antibodies against this epitope during infection and/or vaccination. As it is a single epitope, any antigenic variation in this epitope region could generate false negative results. On the other hand, in FCAT, all antibodies generated against the surface antigens of *L. intracellularis* have accessibility to the diagnostic antigen, and therefore, the sensitivity of the technique is greater. The Indirect ELISA (**Figure 8D**) presents as its main limitation the masking of epitopes, due to the adherence of the bacteria on the plate. An alternative to overcome this limitation is the preparation of biotinylated protein and its immobilization in the correct orientation on streptavidin plates, as described by our group (40). For the development of the latter ELISA, it is first necessary to characterize an immunogenic, specific, and conserved protein; and efforts in this line need to be carried out, although promising results are already available (41).

As already mentioned, the main objective of this study was to develop a method of serological diagnosis for *L. intracellularis* that could be widely disseminated among diagnostic laboratories. Our results demonstrated that the objective was achieved and that FCAT is the most sophisticated immunological technique for the detection of anti-*Lawsonia intracellularis* IgGs. It is important to mention that this technique can be used to detect other classes of antibodies, such as systemic monomeric IgA, IgG1 and IgG2, as well as those associated with the ileum mucosa (data not shown). For this purpose, it is necessary to use specific antibodies (mouse anti-Pig IgG1, clone K139 3C8; mouse anti-Pig IgG2, clone K68 Ig2; mouse anti-Pig IgA, clone K61 1B4, Bio-Rad). The flexibility of FCAT is ideal for studying the immunological profile of licensed vaccines as well as vaccines under development against *L. intracellularis*. Furthermore, as described in **Table 2**, the FCAT is highly accurate, and comparison of precision (intra-assay) and reproducibility (inter-assay) parameters of the other techniques is not available, which makes it difficult to predict the results obtained by the other methods when performed in different laboratories.

A limitation of FCTA, like ELISA, IPMA and IFAT is that it is not possible to differentiate, based on the presence of systemic IgG, vaccinated animals from those naturally infected with *L. intracellularis*. Additionally, one disadvantage of FCAT relative to other diagnostic techniques is the need for the diagnostic laboratory to have a flow cytometer instrument, which is more expensive than an ELISA reader (for the ELISA technique) or a microscope (for the IPMA and IFAT techniques). However, what may be considered expensive today may become affordable in the medium term (such as the situation with polymerase chain reaction machines for example).

We recognize that we have taken an important step towards improving *L. intracellularis* diagnosis, but future studies need to be carried out to develop, on an industrial scale, the other reagents for this diagnosis, such as positive and negative control sera, and conjugated secondary antibodies, which will increase the repeatability of the results and avoid any variations related to the quality and specificity of the commercial reagents (controls and secondary antibodies).

*L. intracellularis*, in addition to produce Ileitis in pigs, also causes an intestinal disease in horses called equine proliferative enteropathy (EPE), which cases have been increasing in the last few years, especially in post-weaning foals and occasionally in adult horses (42). Although the presumptive diagnosis of EPE can be established based on age of the affected animal, clinical signs, and imaging (ultrasonographic evaluation), the confirmation of infection needs to be conducted *in vitro* using molecular or serological techniques. In this scenario, although in this study we did not evaluate FCAT to analyze equine serum, we can speculate, based on existing data on the use of IPMA for the detection of anti-*L. intracellularis* (20, 43), that FCAT has a high potential to work for horses. In this case, future studies need to be carried out to demonstrate the application of this technique in this animal species.

Finally, using the FCAT we analyzed the prevalence of circulating antibodies in a total of 1,000 pigs not vaccinated against *L. intracellularis* from 22 different farms located in 8 Brazilian states. As illustrated in **Figure 7D**, a total of 730 pigs were found to be positive for anti-*Lawsonia intracellularis* IgG; these sera were from animals of 16 different farms (72.7%) and indicates a high prevalence of *L. intracellularis* in Brazilian pig farms. The national prevalence found here is higher than those previously reported in the state of Minas Gerais (37%) (14), which is the fourth main pig producer region of Brazil. Therefore, our results suggest that Brazil is an endemic country for *L. intracellularis*, and that producers should implement biological strategies (vaccines) to mitigate the economic losses caused by this pathogen. Future seroepidemiological studies using the FCAT are needed to better estimate the impact of *L. intracellularis* on Brazilian pig herds.

## Data availability statement

The original contributions presented in the study are included in the article/supplementary material. Further inquiries can be directed to the corresponding author.

## Ethics statement

The animal study was reviewed and approved by Ethics Committee for the Use of Animals in Research at the University of Passo Fundo (CEUA no. 10/2020, 19/2020 & 20/2020). Ethics Committee for the Use of Animals in Research at the Federal University of Minas Gerais (CEUA n° 133/2018. 36/2016).

## Author contributions

Conceived and designed the experiments: RF. Performed the experiments: DZB, JAG, and GCPF. Contributed reagents/materials/analysis tools: RF, LCK, CF, and RMCG. Wrote the paper: DZB, RF, and LCK. Critical review and editing of the manuscript: RMCG, HLW, and SV. All authors discussed the results and commented on the manuscript. All authors contributed to the article and approved the submitted version.
